# Daily administration of low molecular weight heparin increases Hepatocyte Growth Factor serum levels in gynaecological patients: pharmacokinetic parameters and clinical implications

**DOI:** 10.1186/1756-0500-5-517

**Published:** 2012-09-23

**Authors:** Anna Surbone, Luca Fuso, Roberto Passera, Annamaria Ferrero, Cristiana Marchese, Cosimo Martino, Annalisa Luchin, Maria Flavia Di Renzo, Paolo Zola

**Affiliations:** 1 Unit of Gynecologic Oncology, Department of Gynecology and Obstetrics of the University of Torino at Azienda Ospedaliera Ordine Mauriziano, Largo Turati 62, Turin, Italy; 2 Clinical Chemistry Laboratory, Azienda Ospedaliera Ordine Mauriziano, Turin, Italy; 3 Nuclear Medicine Unit, University of Torino at San Giovanni Battista Hospital, Turin, Italy; 4 Department of Oncological Sciences of the University of Torino, Institute for Cancer Research ant treatment Candiolo, Turin, Italy

**Keywords:** HGF, LMWH, Pharmacokinetics, Nadroparin, Epithelial ovarian cancer

## Abstract

**Background:**

Hepatocyte Growth Factor (HGF) enhances cytotoxicity of paclitaxel (PTX) and cisplatin (CDDP) in human ovarian cancer cells. Because of potential pitfalls of HGF exogenous administration, we investigated whether HGF serum concentration might be alternatively raised in vivo by administering low molecular weight heparin (LMWH).

**Methods:**

The main HGF pharmacokinetic parameters were evaluated following acute and chronic LMWH treatment. First, women, operated on for gynaecological tumors, were treated with a single dose of calcium nadroparin and studied for 12 hours. Next, women operated on for benign or malignant gynaecological tumors were treated daily with calcic nadroparin for one month. Subsequently, the biological activity of the measured HGF serum levels was tested in assays of ovarian cancer cell sensitization to drugs.

**Results:**

In the short-term treated group, median HGF AUC_ss_, C_max_ and C_average_ were about four-fold that of the control group, whereas C_min_ was three-fold. In the patients treated chronically median HGF serum levels rose about six-fold in the first week, and decreased but remained significantly higher after one month. The pharmacokinetic of nadroparin-dependent HGF increase were similar in the two groups. The HGF concentrations measured after both acute and chronic treatment were found to be effective in sensitising ovarian cancer cells to chemotherapeutics.

**Conclusions:**

This study raises the possibility of using LMWH to increase HGF serum concentration and to take advantage of its biological activities. In particular, nadroparin might be used as a chemo-potentiating agent in epithelial cell ovarian carcinoma through its action on HGF serum concentration.

**Trial registration:**

ClinicalTrials.gov ID: NCT01523652

## Background

The biological activities of Hepatocyte Growth Factor have been thoroughly investigated both in vitro and in vivo. HGF is implicated in epithelial–to-mesenchymal transition, thus promoting migration, invasion and motility of several cellular types [1]. It promotes blood vessel formation [2] and directs the orderly and homogenous proliferation and organisation of cellular types during embryogenesis, organ regeneration and wound healing [3]. These HGF activities have led to think about therapeutic applications of HGF administration. This growth factor could play a significant role in hepatic regeneration after acute and chronic liver injuries [4-6]*.* Similarly, HGF could be used to facilitate and accelerate regeneration after acute and chronic renal injury [7,8]. After an acute myocardial infarction, HGF shows a cardio-protective action [9]*.*

Several studies have also pointed out the importance of the paracrine action of HGF on neoplastic transformation in several tumor types, such as in the Epithelial Cell Ovarian Carcinoma (ECOC) [10]. The intracellular signalling pathway involving the HGF receptor encoded by the MET oncogene is implicated in cell “invasive growth” [11,12] and the MET oncogene has been found activated in human cancer by over-expression with or without gene amplification, point mutation and autocrine circuit (see at http://www.vai.org/met/). In line with these findings, inhibitors of the HGF-MET axis have been proposed for cancer treatments and are presently undergoing clinical trials. In a preclinical model of ovarian cancer, an orally available small-molecule inhibitor of c-Met, PF-2341066, has been reported to reduce tumour burden and metastasis [13].

However, in 2004, Rasola A. et al. unexpectedly revealed that pre-treatment with HGF enhances the apoptotic response of human ovarian cancer cells to very low doses of paclitaxel (PTX) and cisplatin (CDDP) [14]. Through its receptor and the p38 mitogen-activated kinase (MAPK) pathway [15], HGF acts on the intrinsic pathway activated by the chemotherapeutics in a dose dependent manner. The same research group obtained similar results in a preclinical model into immunocompromised mice [16]: they found a therapeutic window wherein HGF, with a paracrine/autocrine action, sensitises tumours to low doses of CDDP and PTX, which are otherwise ineffective. In this way, they demonstrated that the HGF-mediated enhancement of apoptosis induced by PTX and CDDP lasts even if the cells are exposed to it in a continuous manner.

Following these results, we investigated how the HGF concentration might be raised in vivo. Although, at least hypothetically, it could be possible to use gene therapy or to administer human recombinant HGF (hrHGF), the latter therapies are not standardised and require appropriate studies. We consider an alternative way of raising HGF serum concentration by administering low molecular weight heparin (LMWH) as suggested by preliminary data in the literature [17-19], taking advantage by the fact that heparin is often necessary during the treatment of cancer patients. In the present paper, HGF pharmacokinetic parameters are explored after nadroparin administration in a population of gynaecological patients affected by benign and malignant diseases. We then test HGF effectiveness in sensitising ovarian cancer cell lines to chemotherapeutics at the plasma concentrations obtained in patients following nadroparin administration.

## Methods

### Study protocol

The study consisted of two phases. In the first phase, the main HGF pharmacokinetic parameters were evaluated, comparing a group of six women treated with a single dose of calcic nadroparin to a control group of six untreated women. Venous blood was drawn in both groups at 0, 30, 60, 90, 120, 150, 180, 240, 300, 360, 480 and 720 min. In the second phase, the HGF basal and maximum concentrations were measured in 17 women, undergoing one month of calcic nadroparin daily treatment. Venous blood was drawn twice on day 1 (at 0 and 90 min after nadroparin administration), then once on days 8 and 28 (at 90 min after LMWH injection). Calcic nadroparin was given subcutaneously at 2850 IU/0.3 ml anti-Xa (Fraxiparina®, GSK Italy).

### Patients’ characteristics

In the first phase, 12 patients were enrolled, 6 treated with nadroparin for prophylactic anticoagulation and another 6 untreated as the control group. The six nadroparin-group patients were affected by benign pelvic gynaecologic diseases: three requiring laparoscopy and three laparotomy. All of them were treated at the Gynaecological Oncology Unit of the hospital Azienda Sanitaria Ospedaliera Ordine Mauriziano in Turin, Italy. Their median age was 42 years (range 35–52), and their median BMI was 22.8 kg/m^2^. In the control group, four were healthy women volunteers and two patients submitted to gynaecological pelvic surgery, but these women were not treated with prophylactic LMWH. Their median age was 30 years (range 25–60), and their median BMI was 19.1 kg/m^2^.

In the second phase, 17 patients were enrolled among women planning gynaecological pelvic surgery and treated for 4 weeks with nadroparin for prophylactic anticoagulation. All these patients underwent laparotomy; ten were affected by malignancy (ECOC) and seven by benign (uterine fibroma, ovarian cystadenoma) pelvic gynaecologic diseases. Their median age was 53 years (range 41–75 years), and their median BMI was 22.1 kg/m^2^.

### Inclusion and exclusion criteria

Inclusion criteria for phase 1 were as follows: age ≥ 18 years, ECOG Performance Status ≤ 1; neutrophils ≥ 1500 μl^-1^, platelets ≥ 150000 μl^-1^, creatinine 0.6-1.2 mg/dl, total bilirubin ≤ 1 mg/dl, AST ≤ 35 IU/l, and ALT ≤ 45 IU/l.

Inclusion criteria for phase 2 were the same as for phase one, together with moderate-high risk of deep venous thrombosis (DVT), anticipated anaesthesia > 30 min, obesity, varicose veins, previous DVT and/or pulmonary embolism, thrombophilia, prolonged immobility, and congestive heart failure.

General exclusion criteria were as follows: serious liver or renal diseases, diabetes, hyperlipidemia, serious osteoporosis, pregnancy or lactation, high risk of bleeding (hemorrhagic diathesis, peptic ulcer), inflammatory or infectious diseases, previous cancer, consumption of coffee, tobacco or ethanol in the last 24 hours, and women in treatment with immunosuppressive drugs, contraceptives, lipid lowering agents, or antiaggregants.

All patients and healthy volunteers gave written informed consent prior to enrolment into the study, which was approved by the local ethics committee (CE Interaziendale AO San Giovanni Battista – OIRM Sant’Anna, Torino).

### Reagents and cell line

Cisplatin (CDDP) was from Bristol-Myers Squibb (Rocky Hill, NJ); APC conjugated Annexin V was from Boehringer Mannheim (Indianapolis, IN) and tetramethylrhodamine methyl ester (TMRM) was from Molecular Probes (Eugene, OR); all of the other chemicals were from Sigma (St. Louis, MO). Pure human recombinant HGF was from R&D Systems (Minneapolis, MN). SK-OV-3 cells were purchased from American Type Culture Collection (Manassas, VA) and grown as suggested by the provider.

### HGF determination

The ELISA kit for the determination of HGF in patients’ serum was purchased from BioSource (Camarillo, USA). Blood samples were collected using BD Vacutainer® tubes without additives, blood for serum preparation was left to coagulate and then centrifuged at 3000 rpm for 10 minutes. All samples were coded, stored at −65°C and analysed within 2 months.

### Apoptosis assay

Apoptosis assay was used to evaluate if the HGF concentrations measured in patients after nadroparin administration were effective in sensitising ovarian cancer cells to CDDP. SK-OV-3 ovarian cancer cells were pre-treated with decreasing concentrations of recombinant HGF and, after 48 hours, exposed to medium supplemented with 20 μM CDDP to induce apoptosis. We then measured the percentage of live cells, i.e. cells that did not display either early or late apoptotic features. Flow cytometry recordings of several independent apoptotic changes were performed by a single-tube analysis as previously described (Rasola A. et al., 2004). Briefly, after treatment, cells were resuspended in HEPES buffer (10 mM HEPES, 135 mM NaCl, and 5 mM CaCl2) and incubated for 60 min at 4°C in APC-conjugated Annexin V, TMRM (200 nM), and propidium iodide (PI; 1 μg/ml) to detect phosphatidylserine exposure on the cell surface, mitochondrial inner membrane electrochemical gradient, and plasma membrane integrity, respectively. Cell morphology changes were analysed following variations of the forward and side light scatter. Samples were acquired on a CyAn flow cytometer and analysed with Summit V4.3 software (Dako Colorado, Inc).

### Pharmacokinetic Analysis

The pharmacokinetic study entailed a typical model-free approach, the non-compartmental analysis (NCA). The goal of NCA is to provide an estimate of the main pharmacokinetic parameters by the trapezoidal rule, the only assumption being that the terminal elimination phase can be described by a mono-exponential equation, following first order kinetics. NCA was performed using Kinetica 2000 4.1.1 (InnaPhase Corp., USA), estimating the following HGF parameters: C_max_ (maximum concentration), C_min_ (minimum concentration), C_average_ (average concentration at steady state), T_max_ (time to reach maximum concentration) and AUC_ss_ (area under concentration/time curve at steady state).

### Statistical Analysis

HGF pharmacokinetic parameters were compared during phase 1 (control vs. nadroparin group), phase 1 vs. 2 (nadroparin group) and phase 2 (nadroparin group, benign vs. malignant tumours) by the Mann–Whitney test, while the four HGF levels during phase 2 were compared by repeated measures analysis of variance plus within-subjects contrasts. All reported p values were obtained by two-sided exact method, at the conventional 5% significance level. Data were analysed as of March 2009 by SPSS 17.0 (SPSS Inc., USA). All data are presented as median values and range.

## Results

**Figure 1:**
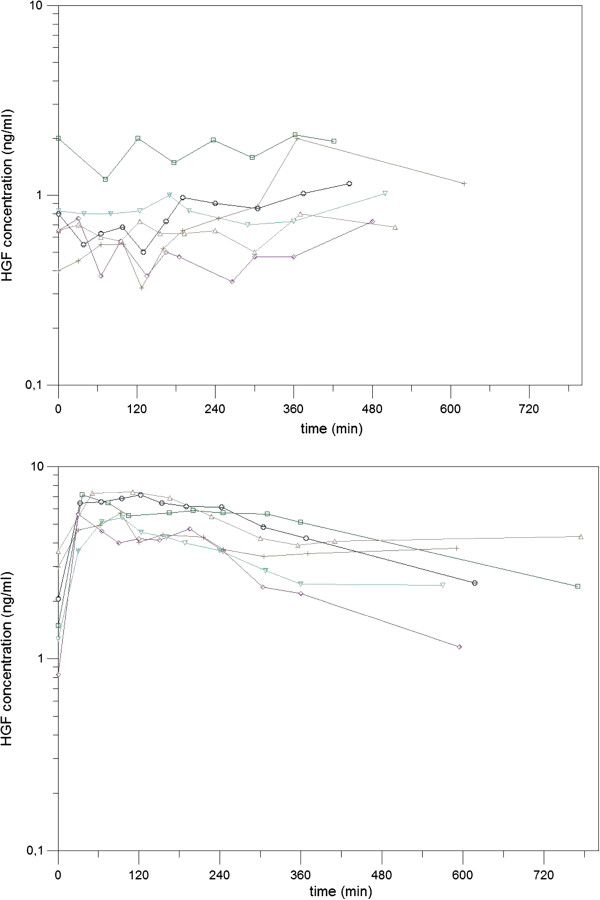
HGF concentration/time curve in phase 1 (controls: 2-5-6-8-10-12; nadroparin: 1-3-4-7-9-11).

The effect of a single nadroparin administration on HGF serum levels was investigated during phase 1 (control vs. nadroparin group); two different HGF kinetics profiles can be recognised at a glance, corresponding to control untreated women and LMWH-treated patients (Figure 1).

Table 1 summarises the main HGF pharmacokinetic parameters, comparing the two phase 1 groups by Mann–Whitney test.

**Table 1:** Median (range) HGF parameters during phase 1, control vs. nadroparin group

**HGF phase_1**	**control**	**nadroparin**	**p**
C_max_ (ng/ml)	1.58 (0.80-5.65)	6.41 (0.75-7.38)	0.087
C_min_ (ng/ml)	0.60 (0.33-1.20)	1.76 (0.35-3.60)	0.037
C_average_ (ng/ml)	0.74 (0.47-2.38)	2.99 (0.34-4.08)	0.065
T_max_ (min)	367 (30–500)	93 (30–122)	0.043
AUC_ss_ (ng*min/ml)	534 (340–1714)	2157 (243–2938)	0.065

In the LMWH group, median HGF AUC_ss_, C_max_ and C_average_ were about four-fold that of the control group, while C_min_ is threefold; at the same time, HGF T_max_ was strongly reduced from about 6 to 1.5 hours, after pretreatment with nadroparin. Data show that nadroparin was able to notably increase HGF serum levels (C_max_ from 1.58 to 6.41 ng/ml), affecting its kinetic behaviour and steady state profile (AUC_ss_ from 534 to 2157 ng*min/ml). The median T_max_ in LMWH group (93 min) can be used as a reference in phase 2 to verify if the HGF concentration’s increase after a single LMWH administration persists even after a month of daily repeated treatment with nadroparin.

**Figure 2:**
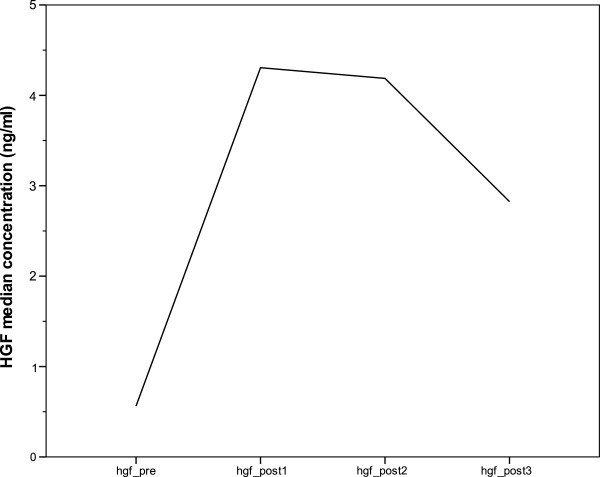
Median HGF concentration during phase 2, nadroparin group.

The 17 patients taking part in phase 2 (nadroparin group, benign vs. malignant tumours) were all treated with daily nadroparin for 1 month. Median HGF serum levels went up from 0.65 ng/ml (basal level at day 0, pre-LMWH administration) to 4.59 ng/ml (C_max_ at day 0, 90 min after LMWH administration). Median HGF C_max_ was almost constant at day 8 (4.19 ng/ml) after the first week of LMWH treatment, whereas at day 28, levels decreased to 2.96 ng/ml; this trend is represented in Figure 2.

From testing HGF C_max_ in phase 1 vs. 2 (both obtained after a single nadroparin administration), the effect on HGF maximum concentrations seems to be minor, even if the statistical significance is borderline (Table 2). In the last period of phase 2 and after 4 weeks of daily LMWH treatment, HGF concentration slightly decreased.

**Table 2:** **Median (range) HGF C**_**max**_** in phase 1 vs. phase 2, nadroparin group**

**HGF phase 1 vs. HGF phase 2**	**HGF phase 1**	**HGF phase 2**	**p**
C_max_ (ng/ml)	6.41 (0.75-7.38)	4.59 (2.75-6.25)	0.060

Basal HGF levels before nadroparin administration (HGF_pre) and HGF C_max_ concentrations after the first nadroparin administration (HGF post_1), after 8 and 28 days of treatment (HGF post_2 and HGF post_3) are represented in Table 3.

**Table 3:** Median (range) HGF levels during phase 2, nadroparin group

**HGF phase 2**	**HGF_pre**	**HGF_post1**	**HGF_post2**	**HGF_post3**	**p**
C_max_ (ng/ml)	0.65 (0.33-1.68)	4.59 (2.75-6.25)	4.19 (1.91-6.08)	2.96 (2.08-5.64)	<0.001
HGF_pre vs. HGF_post1, HGF_pre vs. HGF_post2, HGF_pre vs. HGF_post3					<0.001
HGF_post1 vs. HGF_post2					0.449
HGF_post2 vs. HGF_post3					0.029

Analysing the phase 2 HGF concentrations, it is possible to confirm the HGF pharmacokinetic nadroparin-dependent alteration, already found in phase 1. Using a repeated measures analysis of variance model, the HGF four sequential levels differ from one another (p < 0.001). Applying then the within-subjects contrasts technique enables us to compare the possible couples of phase 2 HGF serum levels. There was a strong, statistically significant difference (p < 0.001) among HGF_pre basal concentrations and any of the three HGF concentrations during nadroparin therapy. The decrease between HGF_post1 and HGF_post2 was moderate (−0.40 ng/ml, p = 0.449), while between HGF_post2 and HGF_post3 was about 30% (−1.23 ng/ml, p = 0.029).

We compared the same previous phase 2 HGF concentrations by stratifying them by the nature of oncological disease in the 17 patients, 10 malignant (ECOC) and 7 benign (uterine fibroma, ovarian cystadenoma) (Table 4). The only statistically significant difference was between HGF_post2 concentrations after the first week of daily nadroparin administration.

**Table 4:** Median (range) HGF levels in phase 2, nadroparin group, benign vs. malignant tumours

**HGF phase_2**	**C_max_ (ng/ml)**	**p**
HGF_pre benign	0.58 (0.38-1.68)	0.584
HGF_pre malignant	0.40 (0.33-1.03)	
HGF_post1 benign	3.70 (2.76-6.19)	0.155
HGF_post1 malignant	4.98 (3.15-6.25)	
HGF_post2 benign	3.18 (1.91-5.73)	0.030
HGF_post2 malignant	5.35 (4.20-6.08)	
HGF_post3 benign	2.96 (2.43-5.64)	0.867
HGF_post3 malignant	2.78 (2.08-4.08)	

In three of our phase 2 patients, we measured basal and Cmax HGF concentration at all the observational points (the first day of heparin treatment, after a week and after a month of daily treatment), and we found that at all points the basal and the Cmax concentrations differed significantly (a median of 0.49 ng/ml at basal concentration versus a median of 3.64 ng/ml at Cmax). Moreover, one of our phase 2 patients was previously under treatment with nadroparin 9500 UI anti-Xa for 11 months because of an episode of deep venous thromboembolism (VTE). In this patient, we observed an HGF concentration increase from 1.15 ng/ml before to 3.84 ng/ml after heparin administration (data not shown).

**Figure 3:**
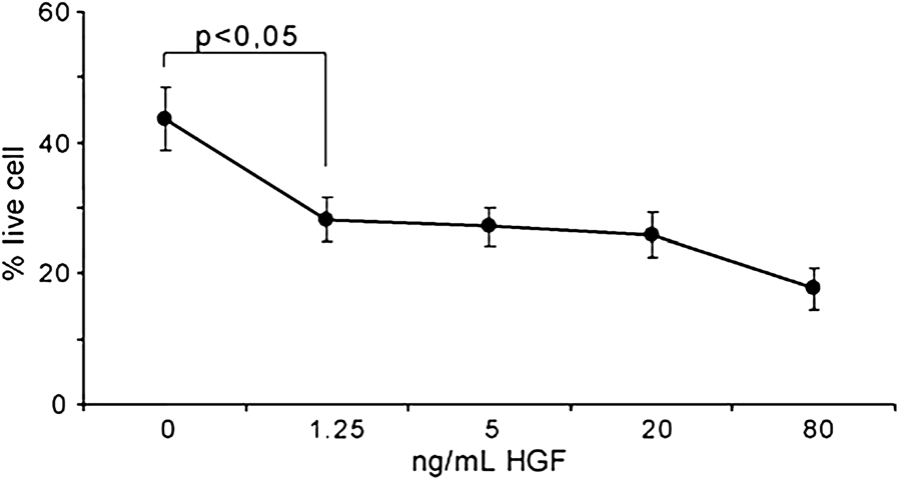
**Dose–response of HGF effects on apoptosis induction by CDDP in vitro**. The effect of HGF on SK-OV-3 ovarian cancer cells is analysed by flow cytometry. SK-OV-3 cells are pretreated for 48 h with HGF at the reported concentrations and then exposed to 20 μM CDDP for 48 h. A representative experiment out of five performed is shown. The Y-axis shows the percentage of live cells, i.e. cells that did not display either early or late apoptotic features. These cells did not express phosphatidylserine on their surface (Annexin V-FITC binding negative), did not display mitochondrial depolarisation (they were not stained with tetramethylrhodamine methyl ester, TMRM) and were not permeable to propidium iodide (PI), as determined using multiparametric fluorescence activated cell sorter analyses.

Finally, we evaluated whether the HGF concentrations measured in our patients after nadroparin administration were effective in sensitising ovarian cancer cells to chemotherapeutics. Rasola A. et al. previously showed that in vitro*,* a concentration as low as 25 ng/ml was effective, but lower concentrations were never tested. Moreover, in a preclinical model, Bardella et al. (2007) demonstrated the strong effectiveness in vivo of a local concentration of 250 ng/ml, extremely higher than that obtained in our patients. We therefore tested the effectiveness of lower decreasing concentrations of recombinant HGF and found that a level as low as 1.25 ng/ml of HGF was able to sensitise ovarian cancer cells to cisplatin (Figure 3).

## Discussion

We studied the pharmacokinetic characteristics of the heparin-induced increase of HGF serum concentration in an attempt to find an alternative way to raise it endogenously rather exogenously. Molecular therapies with HGF have already been devised and seem promising in several diseases. Unfortunately, the exogenous administration of HGF in most cases fails to be effective because of several pitfalls, such as the short half life of the full-size and biologically active HGF, lack of activity of the more stable HGF precursor, which should be processed into the active form, and the poor affinity of shorter HGF analogues, which are biologically active only at high concentrations. Alternative ways of administering the active form have been tested, including the use of plasmid and viral vectors, but, beside the need for standardisation, safety of the HGF therapy should be assessed. For example, in 2005, Nakagami H. et al., showed clinical improvement in 11 patients with critical limb ischemia after the intramuscular injection of naked plasmid DNA of HGF, but biodistribution studies showed that transgene expression was limited to the site of injection.

We studied the increase of HGF serum concentration in vivo after heparin administration. Several studies [20,21] already suggested that some of the biological activities of heparin in vivo, such as boosting angiogenesis and liver regeneration, could be explained by the heparin-induced increase in HGF serum concentration. To take clinical advantage of the HGF-induced activity, avoiding heparin’s anticoagulant activity, it has been proposed [22] the use of decasaccharides produced by the digestion of heparin with heparinase.

Here we report that subcutaneous administration of nadroparin causes a striking and stable increase in HGF serum concentration. In fact, approximately 90 min after a single administration, we obtained a concentration of HGF five-fold that of controls. During an observation time of 12 hours in treated patients, we found an HGF C_average_ four-fold higher than in controls; the elevation in HGF serum concentration reaches a peak after one hour and then decreases progressively, returning to a basal level at about 12 hours after LMWH injection. In the second phase of our study, we measured the HGF serum concentration in patients treated for one month with LMWH, and we found that even at the end of the observations, the elevation in HGF concentration was four-fold higher than before nadroparin injection. In one case, we also measured serum HGF eleven months after daily administration of LMWH because of an episode of deep venous thromboembolism, and we observed that the HGF concentration showed a three-fold increase after heparin administration. Although anecdotal, the latter finding demonstrated that long-term treatment does not cause HGF accumulation and heparin administration is still effective in increasing HGF concentration even after several months of treatment.

In conclusion, we show that heparin induces alterations of the main HGF pharmacokinetic parameters in the short term (as mentioned below) and that these alterations also last after one month of daily treatment with LMWH. Therefore, treatment with calcic nadroparin might be suitable and effective when a clinical and therapeutic use of HGF is anticipated.

We studied gynaecological patients with the intent of exploiting the ability of HGF to sensitise ovarian cancer cells to chemotherapeutic effects. Pre-clinical models showed that HGF enhances the apoptotic effect of low doses of PTX and CDDP on ovarian cancer cells [13-15]. Pre-clinical and in vitro models [13-15] showed that both pre-treatment and continuous exposure of ovarian cancer cells to HGF made them persistently susceptible to drug-induced apoptosis. We demonstrate here that HGF concentrations as low as those obtained after nadroparin injection are effective in sensitising ovarian cancer cells to chemotherapeutics. The combination of these findings encourages further exploration of the possible role of LMWH as a chemo-potentiating agent in ovarian cancer. The possibility of using LMWH in association with chemotherapy (above all, low-doses CDDP and PTX) could be beneficial in the treatment of epithelial ovarian cancer because it could also take advantage of the anti-neoplastic effects of LMWH shown in some studies [23-26] and of its well known antithrombotic action.

The findings reported here are important also when approaching tumour therapy from the perspective of inhibiting HGF and MET, HGF is a Janus-faced molecule, which sensitizes ovarian cancer cells to chemotherapeutics, but is alone able to trigger cancer cell growth and invasiveness. Clinical trials are ongoing, also in ECOC patients, with antibodies and small molecule inhibitor targeting HGF or MET. HGF serum increase after heparin treatment should be taken into account also in this respect.

It has been definitively established that only cancer cells with specific genetic alterations, namely either met gene amplification or HGF autocrine loop maintain dependence on the met gene activation for transformation, i.e. remain met addicted. Therefore, only these cancer cells could respond to met inhibition by small molecule biochemical inhibitors or antibodies. In most instances, HGF and MET axis elicits a number of signalling pathways leading to either cell survival and invasion or cell death or differentiation.

In this study, we tested calcic nadroparin because it is commonly used in our hospital for VTE prophylaxis. Surprisingly, we found that nadroparin causes an elevation in HGF concentration that is higher than most obtained with any LMWH reported in the literature. Salbach PB. et al. [17], for instance, compared the effects of un-fractionated heparin (UFH) and dalteparin on HGF serum concentration in healthy male volunteers. They obtained a similar time course of HGF increase but lower HGF concentration. The main difference between our studies lies in the gender of the patients. Seidel et al. [16] also reported a similarly lower increase of HGF serum concentration following the administration of dalteparin and UFH to three groups of patients treated with different schedules. Only Borawski J. et al. [18] reported a higher increase in HGF concentration, but only after enoxaparin administration in haemodialysed patients. Therefore, it is possible that the striking HGF rise in our patients was due to an amplified responsiveness of patients undergoing surgery and anaesthesia. However, the most likely explanation is that different effects should be attributed to the different kinds of LMWH used in the different studies, which have different pharmacokinetics [27]. There are few studies in the literature comparing the pharmacodynamic profiles of the different LMWHs with respect to HGF, such as that of Rydzewska-Rosołowska et al., who reported that nadroparin, dalteparin and enoxaparin display similar capacity of raising HGF serum concentration [28]. In conclusion, our data show that nadroparin is a good candidate for a possible use as an enhancer of chemotherapeutics because it has excellent bioavailability, a good safety profile and a longer half-life than dalteparin.

The way by which heparin increases HGF serum concentration has yet to be completely clarified. A hypothesis is that heparin could act by removing HGF from its binding to cell surface and matrix proteoglycans [29]. In this way, heparin could either increase HGF clearance from the circulation or take it away from one of its physiologic clearance mechanisms (the reabsorption by the hepatocytes of the HGF-proteoglycans complex) [17]. Heparin can increase the HGF concentration through post-transcriptional regulation mechanisms in the long term. Heparin does not only increase the serum HGF concentration but also increases its activities [30,31] and makes HGF more available in those tissues that need it the most (for instance, ischemia harmed tissues) [17].

## Conclusion

In conclusion, our study opens the possibility of using LMWH to raise HGF serum concentration and take advantage of its biological activities. In particular, we hypothesise that nadroparin could be used as a chemo-potentiating agent in ECOC through its action on HGF serum concentration.

## Competing interest

The authors declare that there are no conflicts of interest.

## Authors' contributions

AS. principal investigator: contributions to conception and design, acquisition of data, analysis and interpretation of data, drafting the manuscript. LF conceived of the study, participated in its design and interpretation of data. RP participated in the design of the study and performed the statistical analysis, Pharmacokinetic Analysis, drafting the manuscript. AF contributions to conception and design of the study. CM contributions to conception and design, supervision of the research group. CM cell lines, apoptosis assay, acquisition of data. AL: ELISA determinations, acquisition of data. MFD participated in its design and coordination and helped to draft the manuscript, revising it critically for important intellectual content PZ. Acquisition of funding, participated in its design, supervision of the research group. All authors read and approved the final manuscript.

## Grant

Fondi per la Ricerca Finanziata dall'Università (ex 60%) 2007.
